# Knowledge and Adherence to Medications among Palestinian Geriatrics Living with Chronic Diseases in the West Bank and East Jerusalem

**DOI:** 10.1371/journal.pone.0129240

**Published:** 2015-06-05

**Authors:** Anas Najjar, Yazan Amro, Islam Kitaneh, Salam Abu-Sharar, Maryam Sawalha, Abrar Jamous, Muhannad Qiq, Enas Makharzeh, Bayan Subb Laban, Wafa Amro, Ahmad Amro

**Affiliations:** 1 Faculty of Pharmacy, Al-Quds University, Jerusalem, Palestine; 2 Faculty of Medicine, Al-Quds University, Jerusalem, Palestine; University of Bologna, ITALY

## Abstract

**Background:**

Adequate patient knowledge about medications is essential for appropriate drug taking behavior and patient adherence. This study aims to assess and quantify the level of knowledge and adherence to medications among Palestinian geriatrics living with chronic diseases and to investigate possible associated socio-demographic characteristics.

**Methods and Findings:**

We conducted a cross-sectional study during June 2013 and January 2014 among Palestinian geriatrics ≥60 years old living with chronic disease in the West Bank and East Jerusalem. A stratified random sample was selected and a questionnaire-assisted interview was applied for data collection. T-test was applied for bivariate analyzing and one-way ANOVA test was applied for multivariate analyses.

**Results:**

A total of 1192 Palestinian geriatrics were studied. The average age was 70.3 (SD=8.58) years and ranged from 60-110 years. The sample comprised 659 (55.3%) females and 533 (44.7%) males. The global knowledge and global adherence scores were (67.57%) and (89.29%), respectively. Adequate levels of knowledge were 71.4%, and of adherence 75%, which were recorded for 705 (59.1%) and 1088 (91.3%) participants, respectively. Significant higher levels of global knowledge and global adherence were recorded for males, and for participants who hold a Bachelor’s degree, those who live on their own, and did physical activity for more than 40 hours/week (p-value <0.05). Furthermore, workers, participants with a higher monthly income, and non-smokers have a higher knowledge level with (p-value <0.05). We found positive correlation between participants’ global adherence and global knowledge (r=0.487 and p-value <0.001). Negative correlation was found between participants’ global knowledge and adherence with age (r= -0.236, p-value <0.001 and r= -0.211 and p-value <0.001, respectively. Negative correlation between global knowledge and the number of drugs taken (r= -0.130, p-value <0.001) was predicted.

**Conclusion:**

We concluded that patients with a higher level of knowledge are more adherent to their medications and that better understanding of socio-demographic factors has a clear influence on the level of knowledge and adherence to medications and thus contributes to the development of guidelines for treatment and may consequently lead to favourable clinical outcomes and savings of health care costs.

## Introduction

Patients are considered to be adherent (compliant) to their medications if they follow the instructions of their health professional [1]. Medication adherence has been studied thoroughly and recognised as a significant issue in treating geriatric patients [2]. Adherence can positively influence treatment efficacy and patient satisfaction as it allows for receiving full benefits of medicine. Moreover, adherence slows disease prognosis by avoiding unwanted complications and decreasing frequent clinical and emergency room visits [3–6]. Conversely, poor or non-adherence to medication has been identified as a significant source of medical errors [2]. This includes decreasing effects of medications and undermines treatment benefits [7]. Moreover, non-adherence causes avoidable morbidity and mortality, higher preventable hospitalization rates and unnecessary loss of health care resources and productivity [8–10].

Patient adherence to medication can be influenced by many factors including the communication pattern between the patient and the health professional, the patient’s cognitive and social beliefs and norms, the patient’s resources including financial, psychological, social support, and the patient’s knowledge [4, 7, 11, 12]. However, ‘knowledge’ has been defined as patient’s awareness of the drug name, purpose, administering schedule, adverse effects, or special administering instructions [13–15]. Adequate patient knowledge about medications is essential for appropriate drug taking behaviour and patient adherence, hence decreasing the use of medical resources, hospital admissions and treatment failure [1, 15].

The incidence of adverse events related to non-adherence has increased among older people [16]. Recent improvement of health care has lead to the rapid growth of the elderly population [17]. This population is often prescribed several drugs to treat chronic diseases which require special care in management and treatment such as hypertension, diabetes mellitus, and pain [18]. Complexity of drug regimens, comorbid conditions, cognitive and psychosocial conditions of older people contribute to the lack of knowledge and adherence in this age group [2, 19]. However, adherence to medication in older people is a poorly understood phenomenon and the most important factors for improving adherence are not yet fully investigated [2].

This study aims to assess and quantify the levels of both knowledge and adherence to medications among Palestinian geriatrics with chronic diseases. Moreover, it aims to investigate possible association between these two factors, together with the patient’s socio-demographic and personal data. It is hoped that understanding of the factors that influence the level of knowledge and adherence, policies and guidelines for treatment could be developed and could ultimately lead to more favourable clinical outcomes and savings of health care costs.

## Materials and Methods

### Study Design

This was a cross-sectional study conducted during June 2013 and January 2014 among Palestinian geriatrics suffering from/ or with chronic disease. Study participants were selected in a stratified random manner. The sample was selected from all governorates (strata) in the West Bank and East Jerusalem according to the size of the population of each. These governorates from south to north are Hebron, Bethlehem, East Jerusalem, Ramallah, Jericho, Tubas, Salfit, Nablus, Qalqilya, Tulkarm and Jenin.

### Study area and population

The West Bank is located at the western side of the Jordan River (32 00 N, 35 15 E). It has a total land area of 5640 km^2^ (including East Jerusalem), and a population of 2,790,331 inhabitants (December 2014) as published by the Palestinian Central Bureau of Statistics (PCBS) http://www.pcbs.gov.ps/.

The study population consisted of all Palestinian geriatrics 60 years of age or older as defined by the PCBS http://www.pcbs.gov.ps/. We selected those who were living with chronic disease/s and in the West Bank and East Jerusalem. Stratified samples representing each governorate were selected. All subjects who were not responsible for their own medication, or suffering from psychological and cognitive disorders and those who were not able to communicate with the investigators were excluded from the study.

### Data Collection

Data were collected through a questionnaire-assisted interview in both English and Arabic. Knowledge about medications and adherence to the prescription were evaluated using validated questions. Each question was read and explained for the elderly person by the investigator to each participant to ensure good understanding of the questions. The answers were recorded by investigators. Each questionnaire was anonymised and given a serial number to facilitate data entry. The questionnaire included four main parts:

#### Personal and socio-demographic data

This includes gender, age, place of residence (governorate), educational level (non schooled, secondary school (Tawjihi) or less, Bachelor's degree, and Master's degree or higher), physical activity (hours/week), marital status (none, divorced, single, married, or widowed), living status (alone, with spouse or relatives, in nursing home, or other), profession (none, working, or retired) monthly household income in NIS (less than 2500, 2500–5000, 5001–7500, 7500+, or refused), smoking status (yes or no), type of insurance (none, governmental, or private).

All subjects were given the choice of refusing to provide any of the previous personal and demographic information, this was expressed by the option ‘none’.

#### Health condition

This includes chronic disease/s reported by each study participant.

#### Drugs section

This includes a list of drug/s used by each participant with measures of patient’s knowledge and adherence for each drug. The variables were categorized into two main categories; **adherence measures** including: Independent administration, expiry status of the drug, method of obtaining “with or without prescription”, and the application of the dose regimen. **Knowledge measures** including: recognition of the drug, knowledge of the drug’s indication(s), mentioning one side effect of the drug, knowledge of proper preservation methods, knowledge of the daily dose, knowledge of how to act when a dose is missed, and knowledge of prescription status. Global knowledge and global adherence were calculated as the average of all knowledge and adherence measures respectively. These measures and calculations were discussed and developed by the authors based on previous studies in the literature [15, 20–22]

### Method of scoring and data analysis

Each knowledge and adherence measure in the questionnaire was given a zero-one score. Positive and negative answers were given 1 and 0 respectively. Score percentages of the seven knowledge measures and the four adherence measures were obtained for each measure/drug and variable separately. An average percentage was then calculated for each measure, later the results were scaled as follows; of the 7 knowledge measures, positively answering 5 (71.4%) was considered adequate representation for sufficient knowledge and positively answering 3 (75%) out of the 4 adherence measures was considered.

Statistical package for social sciences SPSS 16.0 (SPSS Inc., Chicago, IL, USA) was used for data analysis. *T*-test was applied for bivariate analyzing and one-way ANOVA test was applied for the multivariate analyses. The difference between the groups was considered to be significant if *p* < 0.05. Pearson *r* correlation test was applied to measure the degree of the relationship between global knowledge, global adherence, age, and number of drugs used by each patient. The correlation was considered to be significant if *p* < 0.01.

### Ethical considerations

Both the study design and protocols were revised and approved by the Research Ethics Committee at Al-Quds University www.rec.alquds.edu. The study objectives were explained to each participant. Informed consent was obtained in written from each participant. All questionnaires were anonymized and given a serial number to facilitate data entry.

## Results

A total of 1574 Palestinian geriatrics with chronic disease(s) were selected from all governorates in the West Bank and East Jerusalem. Two hundred and fifty eight (16.4%) persons refused to participate and 124 (7.8%) were excluded as they either failed to provide all the data or they defeated parts of the criteria mentioned above. The final sample size was 1192 participants who met the inclusion criteria and took part in the study. The sample size represented (1.15%) of the Palestinian geriatrics population as estimated by PCBS in 2014. The sample comprised 659 (55.3%) females and 533 (44.7%) males. The average age was 70.3 years (SD = 8.6) with a range from 60–110 years. Study participants were selected from each governorate (strata) according to its population size. All governorates, corresponding numbers and percentages of participants are listed in (Table 1).

**Table 1:** Descriptive data calculated for 1192 Palestinian geriatrics living in the West Bank and Jerusalem.

Characteristic	N (%)	Global knowledge %	*p*- value	Global adherence %	*p*-value
**Gender**					
*Male*	533 (44.7)	70.50	<0.001	90.49	<0.021
*Female*	659 (55.3)	65.10		88.32	
**Place of residence**					
*Hebron*	264 (22.1)	62.64	<0.001	87.25	<0.001
*Bethlehem*	111 (9.3)	75.17		87.87	
*East Jerusalem*	190 (15.9)	69.25		88.79	
*Ramallah*	130 (10.9)	63.98		86.69	
*Jericho*	16 (1.3)	70.80		96.34	
*Tubas*	24 (2.0)	69.56		93.57	
*Salfit*	32 (2.7)	67.45		96.20	
*Nablus*	158 (13.3)	69.62		90.56	
*Qalqilya*	34 (2.9)	72.96		93.87	
*Tulkarm*	92 (7.7)	68.01		94.15	
*Jenin*	141 (11.8)	66.83		88.53	
**Education level**					
*Non schooled*	472 (39.6)	60.21	<0.001	85.37	<0.001
*Secondary school (Tawjihi) or less*	572 (48.0)	71.48		91.29	
*Bachelor's degree*	135 (11.3)	75.87		94.25	
*Master's degree or higher*	13 (1.1)	71.35		92.31	
**Type of health Insurance**					
*Governmental*	934 (78.4)	66.96	<0.172	89.73	<0.042
*Private*	89 (7.5)	70.87		85.25	
*None*	169 (14.1)	68.78		89.00	
**Living status**					
*Spouse or relatives*	906 (76)	68.62	<0.001	89.99	<0.001
*Nursing homes*	111 (9.3)	53.93		81.81	
*Alone*	165 (13.8)	70.10		90.30	
*Others*	10 (0.8)	76.31		92.00	
**Physical activity (hours/week)**					
*Less than 1*	203 (17)	64.69	<0.001	89.55	<0.001
*1–20*	533 (44.7)	65.95		87.34	
*21–40*	293 (24.6)	69.45		90.98	
*More than 40*	163 (13.7)	72.66		92.31	
**Profession**					
*None or retired*	996 (83.6)	66.32	<0.001	88.93	<0.052
*Worker*	196 (16.4)	73.57		91.11	
**Monthly income (NIS)**					
*less than 2500*	930 (78)	66.31	<0.001	88.76	<0.060
*2500–5000*	125 (10.5)	76.02		93.35	
*5001–7500*	35 (2.9)	65.75		89.98	
*more than 7500*	22 (1.9)	60.95		88.45	
*refused*	80 (6.7)	70.77		89.07	
**Smoking**					
*Yes*	216 (18.1)	66.44	<0.001	89.15	<0.587
*no*	976 (81.9)	71.91		89.80	

The education levels for study participants were as follows; non-schooled (39.6%), had secondary school (Tawjihi) or less (48%), had a Bachelor's degree (11.3%), and had a Master's degree or higher (1.1%). Most participants had governmental health insurance (78.4%) compared to those who had private insurance (7.5%) or did not have insurance at all (14.1%). Living status of participants showed that (76%) were living with spouse or other family members, (13.8%) were living alone, (9.3%) in nursing homes and (0.8%) reported another living status. However, (61.8%) were married and (38.2%) were widowed, single, or divorced. The average physical activity done by study participants was 19.7 hours/week (SD = 19.7) and (44.7%) ranging from 1–20 hours/week. Table 1 shows number and percentage of participants for each physical activity rang. The majority of participants (83.6%) are non-workers or retired and are nonsmokers (81.9%). The monthly income for (78.0%) of participants was less than 2500 NIS and hence considered as the low-income group (see Table 1).

The average number of drugs per participant was 4.54 (SD = 2.83). The groups of chronic diseases recorded by investigators were mostly cardiovascular, diabetes or painful chronic conditions. Most prevalent diseases were Arthritis (13.7%), Diabetes Mellitus Type 2 (38.2%) and Hypertension (54.3%).

The global knowledge for all knowledge measures scores was (67.51%). The highest score was for the measure (knowledge of proper preservation methods) and lowest was for (mentioning one side effect of the drug) with a score of (92.24%) and (16.87%) respectively. See (Table 2) for all measures ranked from highest to lowest score. Adequate level of knowledge score (71.4%) was recorded for 705 (59.1%) participant.

**Table 2:** Scores of knowledge and adherence measures ranked from highest to lowest score.

Knowledge measures	Score %	Adherence measures	Score %
Knowledge of proper preservation methods	92.24	Expiry status of the drug (i.e. valid drug)	95.28
Knowledge of the daily dose	90.71	Method of correct obtaining i.e. getting the drug with or without prescription	88.63
Knowledge of the drug’s indication	84.4	Independent Administration	87.02
Recognition of the drug	80.55	Application of the dose regimen	86.23
Knowledge of prescription status	59.57		
Knowledge of how to act when a dose is missed	48.24		
Mentioning one side effect of the drug	16.87		
Global Knowledge *	**67.51**	Global Adherence*	**89.29**

*Global adherence and global knowledge were calculated as the average of all adherence and knowledge measures respectively. The cut-off for adequate level of knowledge and adherence is (71.4%) and (75%) respectively.

The personal and socio-demographic characteristics for study participants have shown significantly higher level of global knowledge for males (70.5%), Bethlehem Governorate (75.17%), participants who hold a Bachelor’s degree (75.87%), those who living alone (70.1%), those doing physical activity for more than 40 hours/week (72.66%), workers (73.57%), participants with monthly income of 2500–500 NIS (76.02%) compared to the group of less than 2500 NIS (66.31%), and non-smokers (71.91%) with *p*-value <0.05. However, no significant differences were recorded for the other participant characteristics. (Table 1) summarises all participant characteristics and their corresponding global knowledge scores and *p*-values.

Bivariate correlation analysis showed negative correlation between participant global knowledge and age (*r* = -0.236 and *p*-value<0.001), and the number of drugs taken (*r* = -0.130 and *p*-value<0.001).

The global adherence score was (89.29%). The highest score was for the measure (expiry status of the drug) and lowest was for (application of the dose regimen) with a score of (95.28%) and (86.23%) respectively (see Table 2 for ranking measures). An adequate level of adherence score (75%) was recorded for 1088 (91.3%) participant.

Participant characteristics have shown a significantly higher level of global adherence for males (90.49%), Jericho governorate (96.34%), participants who hold a Bachelor’s degree (94.25%), and who have governmental insurances (89.73%), those living alone (90.3%), and those doing physical activities for more than 40 hours/week (92.31%) with *p*-value <0.05. However, no significant differences were recorded for the other participant characteristics. (Table 1) summarises all participant characteristics and their corresponding global knowledge score.

Bivariate correlation analysis showed positive correlation between participant global knowledge and global adherence (*r* = 0.487 and *p*-value <0.001). However, it showed a negative correlation with age (*r* = -0.211 and *p*-value<0.001), and no correlation with the number of drugs taken.

## Discussion

The extent of knowledge and adherence to medications among Palestinian geriatrics with chronic diseases was assessed in this cross-sectional study. The conducted analyses allowed us to investigate possible associations between the levels of both knowledge and adherence and the patient’s socio-demographic and personal data.

The study design was robust and the selected sample size was adequately representative to the study population as it comprised (1.15%) of the 136,726 Palestinian geriatrics. This was reflected in the even distribution of participants throughout all governorates of the West Bank which allowed collection of enormous and representative socio-demographic and clinical data about Palestinian geriatrics. Moreover, this study was the first of its kind in this area.

The average level of global knowledge for all participants was less than the adequate level previously considered in study design, though (59.1%) of the participants scored an adequate level. This was reflected in knowledge measures and correlated to participant characteristics.

An adequate level was seen for the knowledge measures: recognition of the drug, knowledge of the drug’s indication, knowledge of proper preservation methods, and knowledge of the daily dose. The possible explanation is that the above mentioned information is provided by doctors and pharmacists adequately and are printed or written by the pharmacist on the drug box or package. Moreover, these measures are not too complicated to be understood. This is in concurrence with the education level of the participants as (60.4%) have secondary school level and above. These results are consistent with other studies which concluded that a higher level of education was found to positively affect the aspects of medication knowledge [23–25].

However, an inadequate level was reported for the knowledge measures: knowledge of prescription status, knowledge of how to act when a dose is missed, and mentioning one side effect of the drug which has the least score (16.87%). This could be justified by the complexity of this information which requires a higher education level. This is consistent with the previous study done by Modig *et al*. who found that (60%) of elderly patients knew the indication for their medications while only (6%) knew the risks, side effects, or interactions related to their medications [26]. Moreover, Marks *et al*. reported that 55.8% of the patients were able to state their correct drug names, (93.4%) their dosages, (78.8%) indications, while only (11.7%) were able to mention at least one side effect [26].

An adequate level of global adherence for 1088 (91.3%) participants was reported. This was reflected in the four adherence measures which scored above (75%) hence considered adequate (Table 2). This level of adherence to medications among Palestinian geriatrics is satisfying compared to other countries as reviewed by Di Matteo *et al* and others [4, 27, 28].

Positive correlation between global knowledge and global adherence was predicted. This self-explanatory correlation was described by many previous studies [29, 30]

A Significantly higher level of global knowledge and adherence was reported by males: (70.5%) and (90.49%) respectively. This could be justified by the higher education level of males who had a better chance of education in the past than females. This was reflected in the sample as (81.4%) of males had secondary school or higher compared to (43.4%) of the females. This result contradicts a previous study done in Saudi Arabia which showed no significant differences of knowledge between genders [23]. Another study showed that females are more adherent than males [1]. Moreover, education level has positive association with adherence. This is consistent with other studies since educated people can be more adherent due to their ability to follow instructions related to treatment [31–33].

Knowledge and adherence levels of different governorates showed subtle trends and unexpected results. East Jerusalem was expected to express the highest levels of knowledge and adherence because it has an advanced health care system compared to the West Bank. Further studies are needed to explore and reveal the reasons behind this trend taking into consideration more socio-political and demographic factors.

A significantly higher level of knowledge and adherence was reported for those who live alone, doing 40 hours/week or more of physical activity, having higher knowledge level, and none-smokers. These socio-demographic characteristics indicated higher self-awareness and dependence among these groups which is reflected on their level of knowledge and adherence (authors’ observations). Conversely, a lower level of knowledge was significantly associated with lower income (less than 2500 NIS). This is compatible with other studies which correlated higher income with a higher level of medication knowledge and adherence since participants with higher income had higher education [34]. Moreover, a low socio-economic status is more likely to be accompanied by chronic disease [35, 36]. Furthermore, irregular daily routine, difficult living circumstances, psychological disorders, and unemployment were found to be strongly associated with non-adherence [37, 38]

Negative correlation between the level of knowledge and age was expected. This is in agreement with other studies which concluded that medication knowledge was affected positively by younger age [23, 25]. However, negative correlation between the level of knowledge and the number of drugs taken was revealed. This is consistent with Modig *et al*. who concluded that patients with polypharmacy and multi-dose drug distribution respectively had significantly less knowledge of their medications[26]. Moreover, negative correlation between adherence and participant age is consistent with many studies which showed incomplete adherence for patients below 50 and above 75 years [27, 39].

Participants who have governmental insurance were more adherent. This could be justified by restricted regulations on drug dispensing for governmentally insured patients which possibly influenced patient motivation.

In conclusion, Palestinian geriatrics are adherent to their medication though their average level of medication knowledge is insufficient. Socio-demographic characteristics such as age, gender, or education level are significantly influencing medication knowledge and adherence. Hence, improving patient medication knowledge and will bear a positive effect on medication adherence and better outcome of treatment.
